# Memory B Cell Activation Induced by Pertussis Booster Vaccination in Four Age Groups of Three Countries

**DOI:** 10.3389/fimmu.2022.864674

**Published:** 2022-05-23

**Authors:** Pauline Versteegen, Alex-Mikael Barkoff, Marta Valente Pinto, Jan van de Kasteele, Aapo Knuutila, Sagida Bibi, Lia de Rond, Johanna Teräsjärvi, Katherine Sanders, Mary-lène de Zeeuw-Brouwer, Raakel Luoto, Hinke ten Hulscher, Elizabeth A. Clutterbuck, Elisabeth A. M. Sanders, Jussi Mertsola, Guy A. M. Berbers, Qiushui He, Dominic F. Kelly, Anne-Marie Buisman, PERISCOPE Consortium

**Affiliations:** ^1^ National Institute for Public Health and the Environment, Centre for Infectious Disease Control, Bilthoven, Netherlands; ^2^ Institute of Biomedicine, Microbiology, Virology and Immunology, and Turku University Hospital, University of Turku, Turku, Finland; ^3^ Department of Paediatrics, Oxford Vaccine Group, University of Oxford, Oxford, United Kingdom; ^4^ Department of Paediatric Immunology and Infectious Diseases, Wilhelmina Children’s Hospital, Utrecht, Netherlands; ^5^ Oxford NIHR Biomedical Research Centre, Oxford University Hospitals NHS Foundation Trust, Oxford, United Kingdom

**Keywords:** pertussis, memory B cells, vaccination, longitudinal, children, adolescents, adults, older adults

## Abstract

**Background:**

Immunogenicity of acellular pertussis (aP) vaccines is conventionally assessed by measuring antibody responses but antibody concentrations wane quickly after vaccination. Memory B cells, however, are critical in sustaining long-term protection and therefore may be an important factor when assessing pertussis immunity after vaccination.

**Aim:**

We studied pertussis specific memory B cell (re)activation induced by an aP booster vaccination in four different age groups within three countries.

**Materials and methods:**

From a phase IV longitudinal interventional study, 268 participants across Finland, the Netherlands and the United Kingdom were included and received a 3-component pertussis booster vaccine: children (7-10y, n=53), adolescents (11-15y, n=66), young adults (20-34y, n=74), and older adults (60-70y, n=75). Memory B cells at baseline, day 28, and 1 year post-vaccination were measured by a pertussis toxin (Ptx), filamentous haemagglutinin (FHA), and pertactin (Prn) specific ELISpot assay. Antibody results measured previously were available for comparison. Furthermore, study participants were distributed into groups based on their baseline memory B cell frequencies, vaccine responses were monitored between these groups.

**Results:**

Geometric mean (GM) memory B cell frequencies for pertussis antigens at baseline were low. At 28 days post-vaccination, these frequencies increased within each age group and were still elevated one year post-booster compared to baseline. Highest frequencies at day 28 were found within adolescents (GM: 5, 21, and 13, for Ptx, FHA and Prn, respectively) and lowest within older adults (GM: 2, 9, and 3, respectively). Moderate to strong correlations between memory B cell frequencies at day 28 and antibody concentrations at day 28 and 1 year were observed for Prn. Memory B cell frequencies > 1 per 100,000 PBMCs at baseline were associated with significantly higher memory responses after 28 days and 1 year.

**Conclusions:**

An aP booster vaccine (re)activated memory B cells in all age groups. Still elevated memory B cell frequencies after one year indicates enhanced immunological memory. However, antigen specific memory B cell activation seems weaker in older adults, which might reflect immunosenescence. Furthermore, the presence of circulating memory B cells at baseline positively affects memory B cell responses. This study was registered at www.clinicaltrialsregister.eu: No. 2016-003678-42.

## Introduction

Pertussis can be severe and even lethal for infants, but also patients with (pulmonary) comorbidities and older adults are more prone to hospitalisation and complications (1, 2). The incidence of pertussis has decreased enormously since the introduction of whole cell pertussis (wP) vaccines in the 1940-1950s. However, due to its reactogenicity, wP vaccines have been replaced by acellular pertussis (aP) vaccines in many countries to achieve high vaccination coverage. Despite this high vaccination coverage, a resurgence of pertussis has been noticed in several countries where aP vaccines have been used for routine infant immunisation since the 1990s and where the disease appears to have become endemic again (3–7). The increased circulation may be due to waning vaccine induced immunity, and adaptation of the bacterium. The switch from wP vaccines to aP vaccines for infant priming in high income countries might have influenced this resurgence. In addition, increased awareness and improved diagnostics of pertussis may also have contributed to the increase in the number of reported cases (8, 9).

Recommendations for pertussis booster vaccinations with aP vaccine vary between countries, and may be given at different ages and to different target populations such as pregnant women, military conscripts, healthcare workers and older adults. However, antibody concentrations wane rather quickly after vaccination regardless whether an infant was primed using aP or wP vaccines (10, 11). Despite this, memory B cells may play an important role in long-term humoral immunity, through their mediation of the rapid secondary antibody response following the next encounter with pertussis. Generally, memory B cells reside in the secondary and tertiary lymphoid tissues and are reactivated by antigen encounter with or without T cell help (12). Upon reactivation, memory B cells recirculate throughout the body and may produce either short-lived plasma cells that secrete antibodies, or renewed memory B cells, or long-lived plasma cells. Higher frequencies of antigen specific memory B cells with increasing age have been described in children, which is probably related to vaccination (13, 14). Furthermore, the receipt of even a single dose of a wP vaccine in infants subsequently primed with an aP vaccine, seems to result in a lower risk of infection compared to individuals exclusively aP primed, even several years later (15, 16).

Recently, we have described the serological immunoglobulin (Ig) G antibody concentrations after an aP booster vaccination in four age groups and with different vaccine priming backgrounds in Finland, the Netherlands, and the United Kingdom (UK) indicating a rise in antibody concentrations upon vaccination in all age groups, with substantial inter individual differences in concentrations (10). Differences did not seem to be consistently age-dependent. A subpopulation of this study is now further analysed.

In this study, we identified the frequencies of antigen specific memory B cells before aP booster vaccination, studied the induction of memory B cells one month post-vaccination and monitored the waning of these cells one year post-vaccination. We also assessed potential differences in memory B cell frequencies between four age groups (children, adolescents, young adults, and older adults) within the three study countries (Finland, the Netherlands, and the UK) as well as the effect of previous priming with wP or aP vaccinations on memory B cell frequencies. Furthermore, we evaluated whether the baseline memory B cell frequencies affect vaccination responses.

## Materials and Methods

### Study Design and Participants

**Figure 1:**
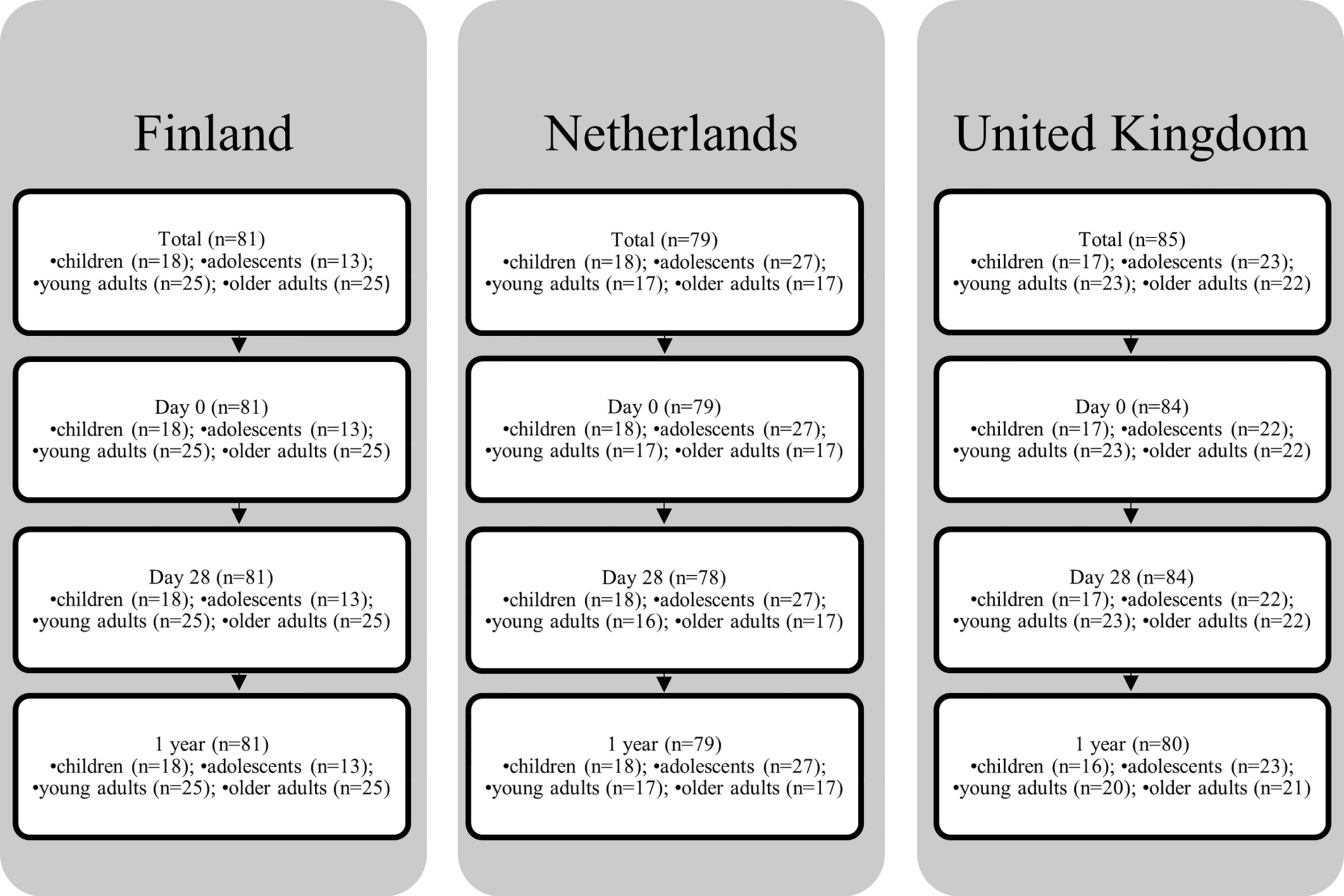
Flow diagram BERT-study.

This phase IV longitudinal interventional study conducted in Finland, the Netherlands and the UK is described in detail elsewhere (10). In summary, healthy children (7-10 y), adolescents (11-15y), young adults (20-34 y), and older adults (60-70 y) received Boostrix-IPV (GlaxoSmithKline (GSK), Wavre, Belgium), a three component pertussis vaccine (Tdap3-IPV), between October 2017 and January 2019. Children were all aP primed, adolescents were either aP or wP primed, young adults were all wP primed, and older adults were either wP primed or unvaccinated. B cell assays were performed on a subset of the total study samples given the resources needed to undertake the assay (total n= 268 out of 379). Samples were selected according to a prespecified sample plan based on participant ID. For the memory B cell analyses the samples were collected before vaccination (baseline), and at day 28 and 1 year post-vaccination. **Figure 1** shows the number of participants per country, per age group, and per timepoint. Their characteristics are listed in **Table 1**. The trial was registered at the EU Clinical Trial database (EudraCT number 2016-003678-42).

**Table 1:** Participant characteristics.

Characteristic		Country	Children aged 7-10 yrs	Adolescents aged 11-15 yrs	Young adults aged 20-34 yrs	Older adults aged 60-70 yrs
No. of participants	Per country.	FI	18	13	25	25
		NL	18	27	17	17
		UK	17	23	23	22
	Total	All	53	63	65	64
Age*	In years	FI	9.0 (8.6-9.5)	13.6 (12.9-14.4)	30.2 (28.7-31.7)	64.2 (63.2-65.2)
		NL	8.5 (8.3-8.7)	13.5 (12.9-14.0)	29.2 (27.2-31.1)	65.6 (64.1-67.1)
		UK	9.3 (8.8-9.8)	12.8 (12.4-13.1)	26.1 (24.1-28.2)	65.6 (64.3-67.0)
Sex^#^	No. female	FI	10 [55.6]	5 [38.5]	21 [84.0]	21 [84.0]
		NL	7 [38.9]	11 [40.7]	6 [35.3]	10 [58.8]
		UK	8 [47.1]	13 [56.5]	15 [65.2]	10 [45.5]
Pertussis primingbackground^$^	No. aP	FI	18 [100]	7 [53.8]	N/A	N/A
		NL	18 [100]	15 [55.6]	N/A	N/A
		UK	17 [100]	23 [100]	N/A	N/A
	No. wP/unknown	FI	N/A	6 [46.2]	25 [100]	25 [100]
		NL	N/A	12 [44.4]	17 [100]	17 [100]
		UK	N/A	0 [0.0]	23 [100]	22 [100]

*Age is indicated in mean with (95% CI). ^#^Sex is indicated in number and [percentage] of female participants per country. ^$^Number of participants aP primed and wP primed in infancy is indicated in number and [percentage] per country. FI, Finland; NL, Netherlands; UK, United Kingdom; No, number; CI, confidence interval; N/A, not applicable for that age group.

### B Cell Analyses

Peripheral blood mononuclear cells (PBMCs) were isolated from whole blood by density gradient centrifugation as described previously with some modifications, as lymphoprep [Axis-Shield (Progen), product no. 1114547] was used instead of vacutainer cell preparation tubes containing sodium citrate (Becton Dickinson Biosciences, CA, USA (BD) (14, 17). PBMCs collected at baseline, day 28, and 1 year post-vaccination were frozen at -135°C after processing, and thawed for memory B cell analyses by antigen-specific ELISpot to run all timepoints per individual simultaneously. After thawing, PBMCs were stimulated for five days using AIM-V medium with AlbuMax (#31035-025, Gibco BRL), 50mM ß-mercapto-ethanol (M3148, Sigma), and 10% fetal bovine serum (FBS) heat-inactivated culture medium supplied with a mixture of CpG ODN 2006 (3 µg/mL), IL2 (10 ng/mL) and IL10 (10 ng/mL). ELISpot plates were coated with pertussis antigens as described previously with minor modifications (14, 17). Briefly, plates were coated with native pertussis toxin (Ptx, 10 µg/mL, GSK), filamentous haemagglutinin (FHA, 5 µg/mL, SP), pertactin (Prn, 5 µg/mL, GSK), and fimbriae types 2 and 3 (Fim2/3, 2.5 µg/mL, GSK) antigens. In addition, PBS was used as a negative control and either anti-human IgG or plasmablasts identification by flowcytometry pre- and post-stimulation served as positive controls. After blocking the wells with culture medium, PBMCs were put on an antigen coated plate with a concentration of 200,000 cells per well, at least in duplicate for 18-24 hours.

PBMC isolation for the memory B cell ELISpot assays were performed at the three different study sites. All study sites used the same validated protocols and same reagents; e.g. the concentration (1:5000) and batch of the secondary antibody (Goat anti-human IgG, Merck-Millipore, 401442-1mL, batch 3167145) were evaluated before the tests to ensure that there is no formation of non-specific spots. ELISpot plates were sent to the Dutch National Institute for Public Health and the Environment (RIVM) and all plates were counted using similar settings at a single ImmunoSpot S6 Ultra-V (Cellular Technology Limited, Cleveland, OH) analyser. Geometric mean (GM) frequencies were calculated for each specific antigen of memory B cells (baseline, day 28, and 1 year post-vaccination) per 100,000 PBMCs and the background frequency from the negative control was subtracted per individual. All memory B cell frequency data have been deposited in the central database of the PERISCOPE Consortium and can be accessed by a request to the PERISCOPE management team.

### Statistical Analysis

For statistical analysis, a frequency of 0 memory B cells was replaced by 0.1. For the memory B cell frequencies, a linear mixed model was fitted to the log-transformed frequencies for each memory B cell frequency and antigen combination. A linear mixed model can be considered as a generalisation of a paired t-test (18). This model describes the GM of memory B cell frequencies while accounting for the longitudinal structure of the measurements. Timepoint of blood sampling, age group, and country were included in the model as a three-way interaction as fixed effects. Sex was included only as main effect. Participant ID was included as a random intercept in the model. This took the within-participant correlation into account. A similar model, where age group was replaced by vaccination group, was used to analyse the differences in frequencies between individuals with an aP or wP primary vaccination background.

A type III ANOVA was used to assess overall significance of the fixed effect terms. GM frequencies and their corresponding 95% confidence intervals (95% CI), as well as their mutual GM frequency ratios with corresponding 95% CI, and p-values were obtained by *post hoc* analysis. Satterthwaite’s method was used for the mixed model denominator degrees of freedom (19, 20). P-values were adjusted by applying the Benjamini-Hochberg procedure for multiple comparisons, controlling the false discovery rate (21).

Statistical analysis were done in R, using the lme4 package, and emmeans package (22–24).

Data regarding antigen specific IgG antibody concentrations within the study groups have been published previously (10). In the current study, we correlated these antibody data to specific memory B cell frequencies in the total study cohort. Pearson’s r was calculated for the log10 transformed data using GraphPad Prism 9.0.1 for Windows. Based on the results, heat maps were created. A correlation of exactly 1 is considered perfect, ≥ 0.70 as strong, ≥ 0.50 as moderate, and <0.30 as weak.

To identify a possible biomarker of a proper memory B cell vaccine response, cut-off values for baseline frequencies were determined by dividing the participants into ordinal groups with the following baseline GM memory B cell frequencies: ≤1, >1-2, and >2 per 100,000 PBMCs. These groups were compared to reject the null hypothesis that there is no significant difference between the groups at any timepoint (baseline, day 28, and 1 year post-vaccination). Same cut-off was also used to present the antibody GM concentrations (GMCs) of the two groups. Data analysis was performed using IBM SPSS Statistics for Windows, Version 25.0.0.0 (IBM Corp., Armonk, NY, USA).

## Results

### Memory B Cell Frequencies in Each Specific Age Group at Different Timepoints

**Figure 2:**
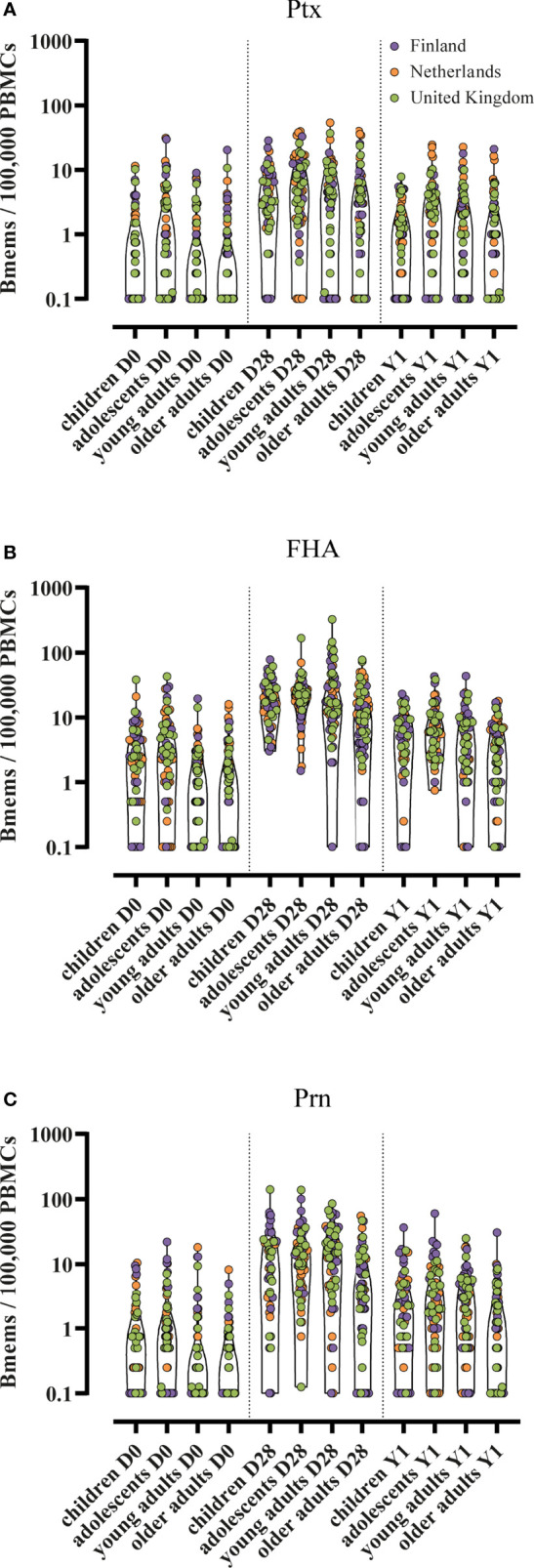
Individual memory B cell frequencies in four age groups. Bmems, memory B cell frequencies; PBMCs, peripheral blood mononuclear cells; D0, day 0 (baseline); D28, day 28 post-vaccination; Y1, 1 year post-vaccination. Memory B cell frequencies specific for **(A)** pertussis toxin; **(B)** filamentous haemagglutinin; and **(C)** pertactin. Presented in truncated violin plots. Corresponding geometric mean frequencies and 95% confidence interval are presented in **Table 2**.

In this study, a total of 268 healthy participants across Finland, the Netherlands and the UK were included and received a Tdap3-IPV booster vaccination: children (7-10y, n=53), adolescents (11-15y, n=66), young adults (20-34y, n=74), and older adults (60-70y, n=75). Longitudinal samples were available from most participants, however in a small proportion of participants one or more timepoints were missing (**Figure 1**). The median spot frequency in the blanc wells was 0/100,000 cells (95% CI 0-0) and the mean spot frequency was 0.21/100,000 cells (95% CI 0.18-0.23). A scan of a representative plate showing spot frequencies per antigen is available in **Supplementary Figure 1**. Generally, only minor differences were noticed in memory B cell frequencies between the three countries within each age group (**Figure 2** and **Supplementary Table 1**). When age groups were combined from all countries, baseline GM memory B cell frequencies were all > 0.1 per 100,000 PBMCs (**Table 2**). However, on an individual level the majority of participants had memory B cell frequencies of 0.1 per 100,000 PBMCs to either Ptx, and/or FHA, and/or Prn at baseline (**Table 2**). At day 28 post-vaccination we noticed significantly higher GM memory B cell frequencies within each age group for Ptx, FHA and Prn (p ≤ 0.001 for all, **Table 3**. However, in each group we found non-responders varying from 4.6% to 37.5% of all participants (**Table 3**). The proportion of non-responders was usually highest among older adults. At 1 year post-vaccination significantly lower GM memory B cell frequencies were observed when compared to 28 days post-vaccination for all antigens among all age groups (p ≤ 0.001 for all). GM memory B cell frequencies 1 year post-vaccination were still significantly elevated compared to baseline within all age groups and for all antigens (p values ≤ 0.001-0.05), except against Ptx in children. The GM frequencies of Ptx specific memory B cells were relatively low at each timepoint, compared to those for FHA and Prn. Memory B cell frequencies for Fim2/3 (pertussis antigen not in the vaccine) have not been included in the analyses as they were mainly low in all age groups at all timepoints (data presented in **Supplementary Figure 2**).

**Table 2:** Antigen specific geometric mean memory B cell frequencies per age group with 95% confidence intervals and significances from all countries together were calculated using a linear mixed model on the log10 transformed data taking the longitudinal structure into account.

Antigen	Timepoint	Children	Adolescents	Young adults	Older adults
Ptx GM Bmem frequencies /100,000 PBMCs (CI)	day 0	0.5 (0.4-0.8)	0.9 (0.6-1.3)^a,b^	0.4 (0.3-0.6)^c^	0.4 (0.3-0.6)^c^
	day 28	3.4 (2.2-5.1)	4.9 (3.3-7.3)^a,b^	2.5 (1.7-3.6)^c^	2.0 (1.3-2.9)^c^
	1 year	0.8 (0.6-1.3)	1.4 (1.0-2.1)	1.0 (0.7-1.5)	0.8 (0.5-1.2)
FHA GM Bmem frequencies /100,000 PBMCs (CI)	day 0	2.1 (1.5-3.0)^a,b^	2.9 (2.1-4.0)^a,b^	0.7 (0.5-1.0)^c,d^	0.8 (0.6-1.0)^c,d^
	day 28	18 (13-25)^b^	21 (15-29)^b^	19 (14-25)^b^	8.7 (6.4-12)^a,c,d^
	1 year	4.2 (3.0-5.8)^b^	6.7 (4.9-9.3)^a,b^	3.7 (2.7-5.0)^c^	2.4 (1.7-3.3)^c,d^
Prn GM Bmem frequencies /100,000 PBMCs (CI)	day 0	0.6 (0.4-0.9)^a,b^	0.8 (0.5-1.1)^a,b^	0.4 (0.3-0.5)^c,d^	0.3 (0.2-0.5)^c,d^
	day 28	8.1 (5.5-12)^b^	13 (8.6-18)^b^	9.2 (6.4-13)^b^	3.0 (2.1-4.3)^a,c,d^
	1 year	1.8 (1.2-2.6)^b^	2.7 (1.9-4.0)^b^	1.8 (1.3-2.7)^b^	0.8 (0.6-1.2)^a,c,d^

Significance per antigen has been tested between age groups within a timepoint; p ≤ 0·05. Significantly different from, ^a^young adults; ^b^older adults; ^c^adolescents; ^d^children. Ptx, pertussis toxin; FHA, filamentous haemagglutinin; Prn, pertactin; GM, geometric mean; Bmem, memory B cell; PBMC, peripheral blood mononuclear cells; CI, 95% confidence interval.

**Table 3:** Distribution of participants. At day 0 (baseline), the amount of participants with memory B cell frequencies ≤ 1/100,000 PBMCs are shown.

Antigen	Group	Day 0 ≤ 1 spot/100,000 PBMCs (%)	Day 28 ≤ 1 spot/100,000 PBMCs and/or ≤ day 0 (%)	1 year ≤ 1 spot/100,000 PBMCs (%)
Ptx	**Total**	**169 (69.3)**	59 (24.4)	117 (48.8)
	Children	35 (66.0)	10 (19.2)	27 (51.9)
	Adolescents	36 (58.1)	9 (14.8)	23 (36.5)
	Young adults	49 (75.4)	18 (27.7)	29 (46.8)
	Older adults	49 (76.6)	24 (37.5)	38 (60.3)
FHA	**Total**	**103 (42.2)**	15 (6.2)	38 (15.9)
	Children	15 (28.3)	3 (5.8)	6 (11.5)
	Adolescents	16 (25.8)	3 (4.9)	2 (3.2)
	Young adults	38 (58.5)	3 (4.6)	9 (14.8)
	Older adults	35 (54.7)	6 (9.4)	21 (33.3)
Prn	**Total**	**184 (75.4)**	35 (14.5)	90 (37.5)
	Children	36 (67.9)	7 (13.5)	18 (34.6)
	Adolescents	41 (66.1)	3 (4.9)	17 (27.0)
	Young adults	52 (80.0)	7 (10.8)	21 (33.9)
	Older adults	55 (85.9)	18 (28.1)	34 (54.0)
Ptx, FHA, & Prn	**Total**	**73 (29.9)**	4 (1.7)	23 (9.6)
	Children	11 (20.8)	–	2 (3.8)
	Adolescents	10 (16.1)	–	1 (1.6)
	Young adults	21 (32.3)	–	5 (8.2)
	Older adults	31 (48.4)	4 (6.3)	15 (23.8)

At day 28 post-vaccination the amount of non-responders is shown, defined as a frequency of ≤ 1 memory B cell/100,000 PBMCs and/or ≤ day 0. At 1 year post-vaccination again the participants with memory B cell frequencies ≤ 1/100,000 PBMCs are shown. Data are shown per antigen and for the combined antigens, for the total group as well as split per age group. The total group per antigen at baseline (bold) shows the distribution of participants used for **Figure 5**. Ptx, pertussis toxin; FHA, filamentous haemagglutinin; Prn, pertactin; PBMCs, peripheral blood mononuclear cells.

### Memory B Cell Frequency Differences Between Age Groups

GM memory B cell frequencies specific for Ptx detected at baseline were < 1 per 100,000 PBMCs for all age groups (**Table 2**). At day 28 post-vaccination, Ptx specific memory B cell frequencies were low, but significantly higher in adolescents (GM 4.9) compared to young (GM 2.5, p < 0.05) and older adults (GM 2.0, p ≤ 0.01). At 1 year post-vaccination, significant differences were lost between the age groups and the GM frequencies of memory B cells were overall low. In general, adolescents had the highest GM frequency of Ptx specific memory B cells and older adults the lowest.

GM frequencies of specific memory B cells against FHA were the highest compared to the other two pertussis vaccine antigens among all age groups. At baseline, GM memory B cell frequencies were low among all age groups, although slightly higher in children and adolescents compared to young and older adults. At day 28 post-vaccination, children, adolescents and young adults (GM 18, 21, and 19, respectively) had higher frequencies of memory B cells compared to the older adults (GM 8.7, p ≤ 0.01, p ≤ 0.001, and p ≤ 0.001 respectively). At 1 year post-vaccination significant differences were observed between children and older adults, between adolescents and young adults, and between adolescents and older adults. In general, adolescents had the highest GM frequencies of FHA specific memory B cells and older adults the lowest.

Prn specific GM memory B cell frequencies at baseline were < 1 per 100,000 PBMCs for all age groups. At day 28 post-vaccination frequencies were significantly higher in children, adolescents, and young adults (GM 8.1, 13 and 9.2 respectively), compared to older adults (GM 3.0, p ≤ 0.001). One year post-vaccination, children, adolescents, and young adults still had higher frequencies of Prn specific memory B cells. In general, the Prn specific GM memory B cell frequency was the highest among adolescents and the lowest among older adults.

### Adolescents With Different Vaccination Background

**Figure 3:**
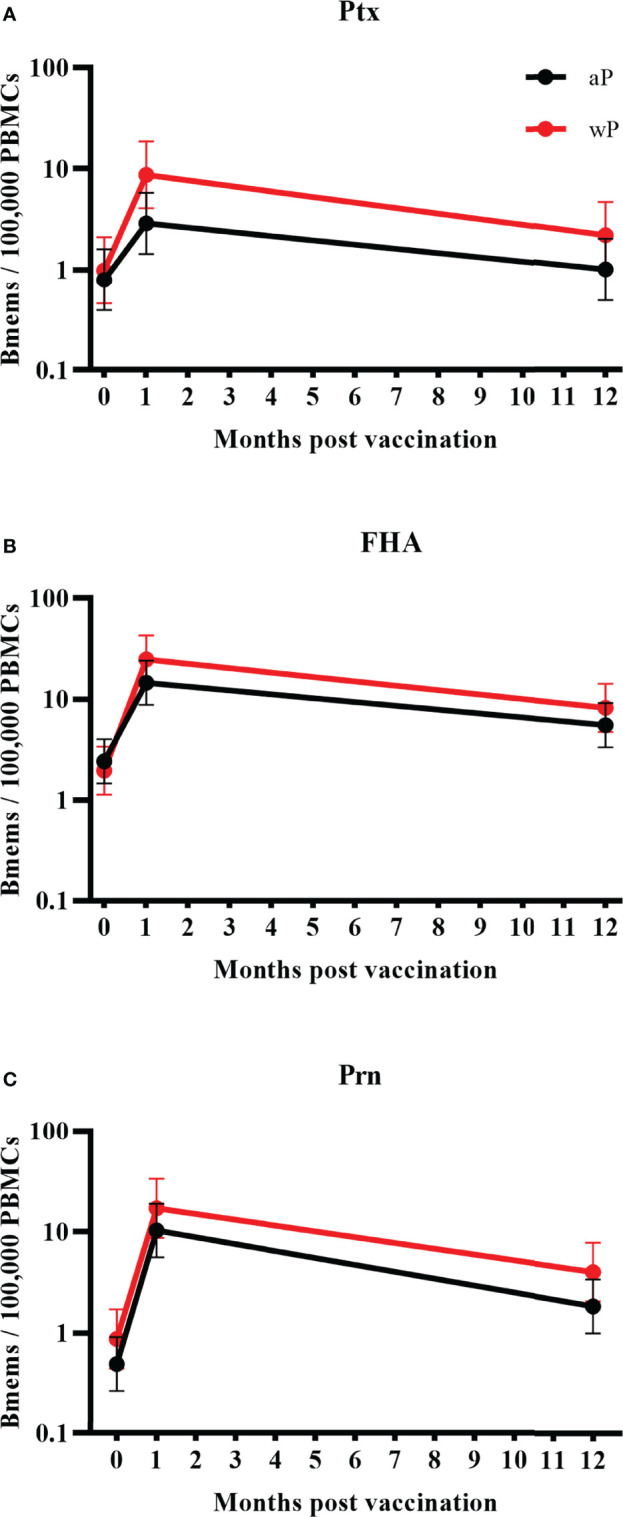
Geometric mean memory B cell frequencies per priming vaccination background with 95% confidence intervals. Significances were calculated using a linear mixed model on the log10 transformed data taking the longitudinal structure into account. Bmems, memory B cell frequencies; PBMCs, peripheral blood mononuclear cells. Memory B cell frequencies in adolescents from the Netherlands and Finland together (aP priming n=22, wP priming n=18) specific for **(A)** pertussis toxin; **(B)** filamentous haemagglutinin; and **(C)** pertactin. Observed differences between the aP and wP primed cohorts did not reach significance.

Adolescents from Finland and the Netherlands were primed with either aP (n=22) or wP (n=18) vaccine. We observed higher GM memory B cell frequencies within the wP group compared to the aP group for all antigens at all timepoints, excluding FHA at baseline (**Figure 3**). At day 28 post-vaccination differences were most pronounced between the two groups, however not significant. At 1 year post-vaccination, GM memory B cell frequencies for wP primed individuals were still 1.5 to 2.2 fold higher when compared to those of the aP primed individuals. This comparison was not available in the UK since all adolescents were aP primed as per UK national immunisation programme.

### Correlation Between Memory B Cell Frequencies and IgG Antibody Concentrations

**Figure 4:**
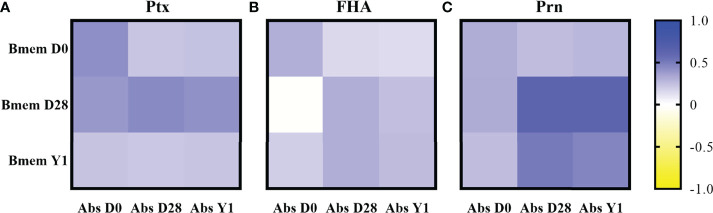
Correlations between memory B cell frequencies and antibody concentrations presented as heatmaps per antigen for all participants together based on Pearsons’ correlation on the log10 transformed memory B cell frequencies and antibody concentrations. Ptx: pertussis toxin; FHA, filamentous haemagglutinin; Prn, pertactin; Bmem, memory B cell frequencies; Abs, antibody concentrations; D0: day 0 (baseline); D28, day 28 post-vaccination; Y1, 1 year post-vaccination. Correlations for **(A)** pertussis toxin; **(B)** filamentous haemagglutinin; and **(C)** pertactin.

Correlations between memory B cells frequencies and IgG antibody concentrations against different antigens can be found in **Figure 4**. For all participants together at day 28 post-vaccination, the correlation between antigen specific memory B cell frequencies and IgG antibody concentrations was moderate/strong for Prn (r = 0.62, p < 0.001), whereas those for Ptx and FHA were weak/moderate at the same timepoint. At 1 year post-vaccination, weak to moderate correlations were noticed for all antigens, still showing the highest correlation for Prn (r = 0.46, p < 0.001). Prn specific memory B cell frequencies at day 28 post-vaccination correlated also moderate/strong with Prn specific antibody concentrations 1 year post-vaccination (r = 0.62, p < 0.001), whereas those correlations for Ptx and FHA were again weak/moderate. Generally, correlations for FHA were the lowest compared to Ptx and Prn. We additionally measured antigen-specific IgG producing plasma cell frequencies at day 7 post-vaccination, also showing the highest correlation to memory B cell frequencies and antibody concentrations at day 28 and 1 year post-vaccination for Prn. Plasma cell methods and results are supplied in **Supplementary Figure 3**.

### Memory B Cell Frequencies at Baseline as a Marker of Vaccine Responsiveness

**Figure 5:**
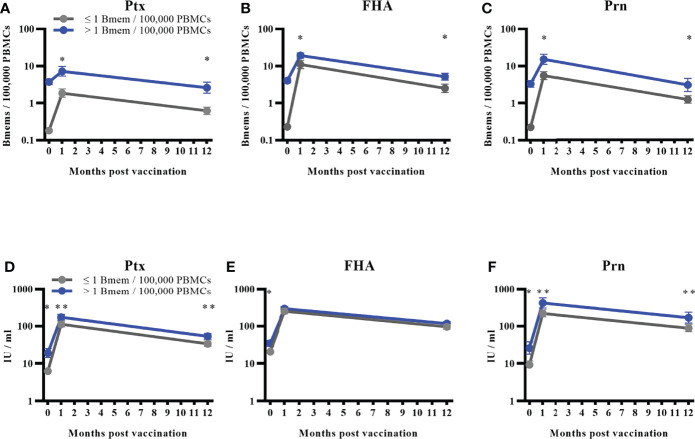
Memory B cell responses to different antigens based on a defined frequency cut-off of > 1 memory B cell/100,000 PBMCs. Bmems: memory B cell frequencies; PBMCs, peripheral blood mononuclear cells. Geometric mean memory B cell frequencies with 95% confidence intervals for the combined age groups distributed into two groups based on baseline memory B cell frequencies for **(A)** pertussis toxin; **(B)** filamentous haemagglutinin; and **(C)** pertactin. Corresponding antibody geometric mean concentrations for the two groups for **(D)** pertussis toxin, **(E)** filamentous haemagglutinin; and **(F)** pertactin. Number and proportion of participants in the group with frequencies ≤ 1 memory B cell/100,000 PBMCs can be viewed in **Table 3**. *p < 0.001; **p = 0.003.

We studied the influence of baseline memory B cell frequencies on vaccine responses against all pertussis vaccine antigens. Study participants from all age groups of the three countries were distributed into two categories based on the previously mentioned ordinal groups. We defined a frequency cut-off of > 1 memory B cell per 100,000 PBMCs at baseline for Ptx, FHA, and Prn, which was the lowest frequency to show significant difference between the two groups. This is on average 5 times higher compared to the mean background value. These cut-offs resulted in a rather even distribution of participants within both categories (**Table 3**). Results of GM memory B cell frequencies post-vaccination in these two categories are presented in **Figures 5A–C**. Corresponding antibody GMCs at all timepoints from the two categories are displayed in **Figures 5D–F**).

Significantly higher GM memory B cell frequencies were observed at day 28 and 1 year post-vaccination in individuals showing a frequency of > 1 memory B cell per 100,000 PBMCs compared to individuals showing a frequency of ≤ 1 memory B cell per 100.000 PBMCs at baseline for all three vaccine antigens (p < 0.001 for all). For antibodies, significantly higher GMCs were observed at baseline for all three antigens (p < 0.001) and these remained significant for Ptx and Prn (p = 0.003 at day 28 and 1 year) in individuals having memory B cells > 1 per 100,000 PBMCs at baseline. For FHA, the two groups were not significantly different at day 28 and 1 year post-vaccination as could be expected from the weak correlation between memory B cell frequencies and antibody concentrations.

## Discussion

In this study, we measured antigen (Ptx, FHA, and Prn) specific memory B cell frequencies within four age groups in three countries after an aP booster vaccination. At baseline GM memory B cell frequencies were found to be low among all age groups, whereas a significant increase in memory B cell frequencies was found one month post-vaccination, to the three pertussis vaccine antigens in all age groups. The percentage of non-responders varied from 4.6 to 37.5%, with highest proportion of non-responders in older adults. Furthermore, memory B cell frequencies were still significantly elevated one year post-vaccination compared to baseline, indicating a persistent response following aP booster vaccination at all ages from 7 to 70 years of age. Generally, all age groups within the three countries showed similar patterns in memory B cell frequencies with minor country to country variations. However, variation was observed between the age groups. The lowest post-vaccination frequencies were constantly observed within the older adults (60-70 years), whereas the highest frequencies were among the adolescents (11-15 years). Furthermore, the older adults had lower memory B cell frequencies compared to the young adults (20-34 years). In addition, the presence of > 1 memory B cell per 100,000 PBMCs at baseline affects the memory B cell responses positively.

As stated, the highest post-vaccination memory B cell frequencies were found in adolescents. The infection pressure experienced by the different age groups might partly explain this. In 2017/2018, ECDC reported higher age-specific notification rates per 100,000 in adolescents compared to school-aged children and adults for all three study-countries (25). In a Dutch population-based sero-surveillance study performed in 2016-2017, infection prevalence based on serum antibodies was highest among adolescents (11.5%), followed by school aged children and lower among adults (6). Interestingly, this trend is similar to that of the frequencies of memory B cells found in this study. Another sero-survey performed among European young adults in 2011-2012 confirms these data and showed a low proportion of infected young adults (1.6%) in Finland (26). Although, the study period of these studies differed, they both show the general trend of naturally circulating *B. pertussis* in these populations. Memory B cell frequencies among adolescents were slightly higher (but not significant) compared to that in children. This is in line with a previously published study among wP primed 3-9-year-olds by Hendrix et al, 2011 (13). They showed increasing memory B cell frequencies with increasing age, however the difference between 6- and 9-year-olds was not significant which is consistent with the findings in the current study where adolescents and children had similar frequencies of memory B cells in the circulation. In this study, the older adults had significantly lower post-vaccination memory B cell frequencies compared to the other age groups. This contrasts to the previously measured IgG antibody concentrations one month and one year post-vaccination, that were not uniformly less in older adults (10). However, lower immune responses in older adults have been previously described, and the explanation for this is probably multifactorial (27, 28). The first aspect to be considered in this age group is immunosenescence (28). This phenomenon leads to weaker immune responses after an encounter with a specific stimulus in comparison with other age groups, involving both B and T cells. It is also known to compromise vaccine responses (29). The B cell recall response is lower in older adults whereas aged memory B cells are less capable of differentiating into plasma cells upon boosting (28, 29). This might also be applicable for *in vitro* stimulation of memory B cells to plasmablasts since aged memory B cells might respond less to CpG stimulation (30). Also, the majority of the older adults comprised of individuals who have not been primarily immunised with a pertussis vaccine. However, these individuals have probably gained their primary protection against pertussis from natural infection and are probably boosted by infection multiple times in their lives (29). Previously reported antibody concentrations measured in the participants of the current study, reported higher IgA antibody concentrations in the older adults compared to the other age groups (10). This suggests that part of the protection in older individuals is guided towards IgA instead of IgG (29). A further consideration for the older adults group is that repeated exposure through carriage or infection to *B. pertussis* involves specific forms of antigen which may differ in their epitope content from those antigens in aP vaccines (or those purified antigens used in assays) and result in a less effective memory response to the aP booster vaccination (31–33).

This study included participants with different vaccination backgrounds as published before by Versteegen et al., 2021 (10). It is well known that wP vaccines contain a broad repertoire of low dose bacterial components whereas aP vaccines contain a relatively high dose of only a subset of (chemically detoxified) antigens. Immunisation using wP vaccines seems to induce immunity against the pathogen, whereas aP vaccines induce immunity against the vaccine antigens. Therefore, wP primed individuals will probably produce more antibodies when boosted by natural infection where aP primed individuals will probably produce more antibodies when boosted by aP booster vaccines (31). However, we noticed a trend to higher memory B cell frequencies against the three measured vaccine antigens in wP compared to aP primed adolescents. One possible explanation could be the different intervals since the latest booster vaccine, as the wP primed individuals received their previous booster dose approximately two (the Netherlands) or a half (Finland) years earlier compared to their aP primed peers. In contrast to our findings, higher memory B cell frequencies have been shown in boosted 4 year old aP primed compared to wP primed individuals (34). Interestingly, a Dutch study performed among children 9 years of age, showed that after a second aP booster at 9 years of age, wP primed individuals had higher frequencies of memory B cells than aP primed children (11). Our results provide more support that a second aP booster results in opposite findings compared to a first booster as we observed higher frequencies of circulating memory B cells in wP primed adolescents aged 11-15 years who received their second booster. Some previous studies have shown that long-term protection against infection after wP priming, even when mixed with aP boosters, is better compared to just aP priming (15, 16, 31, 35). For our analysis, the small number of participants for this comparison limited the power to detect a significant difference. However, significant differences between aP and wP primed individuals have been reported in a subset of the study participants using high dimensional flow-cytometric analyses showing significantly stronger IgG1+ plasma cell responses in wP primed individuals, which was the most prominent subclass at day 7 (36). Also stronger Th2 responses were reported in aP primed versus wP primed individuals (37). Stronger Th2 responses in aP primed individuals have been described often, in young children as well as in adolescents (38). Therefore, age difference does not seem to explain why aP primed young children have better B cell responses following an aP booster while in adolescence wP primed individuals have higher frequencies. However, it is possible that the amount of aP boosters might be of more influence on this issue. A group of wP primed young adults (20-34 years) was also included in this study. Results from this group indicated a lower frequency of memory B cell frequencies in young adults compared to adolescents, but similar or even higher frequencies compared to the children’s group. Most young adults have never received additional pertussis booster vaccinations, which may indicate that wP priming is more efficient than aP priming in long-term immunity (39). However, it is also possible that these individuals have had their immunity boosted by mild infections from time to time (6, 40). To strengthen this aspect, a recently published seroprevalence study among adults in childbearing age (20-39 years) showed clear circulation of pertussis in this age group in several European countries (26).

We demonstrated best correlation between memory B cell frequencies and long-term antibody concentrations for Prn. Jahnmatz et al., 2014 compared B cell responses to humoral immunity after a fifth dose of a single component pertussis vaccine (TdAP1, 20 µg PT) versus a five component pertussis vaccine (TdaP5, 2.5 µg PT, 5 µg FHA, 3 µg Prn, and 5 µg Fim2/3). They showed a good correlation between antibody concentrations and memory B cell frequencies to Ptx with the high dose AP1 vaccine. However, with the low dose aP5 vaccine, there was a poor correlation for Ptx, FHA and Prn (41). The latter finding is quite similar to our findings (excluding Prn), whereas a relatively low dose of a three component pertussis vaccine (TdaP3-IPV, 8 µg PT, 8 µg FHA, and 2.5 µg Prn) was used in this study. This may either suggest that B cell memory is enhanced less efficiently after a multi-component aP booster, or by the lower dose of the antigens included. It has been shown that FHA and Prn are both immunogenic (42, 43). Studies by Jahnmatz et al., 2014 and Schure et al., 2013 show that antibody concentrations post aP booster are higher for Prn than for FHA in children, whereas in our study groups (all except older adults), these values were close to each other (10, 41, 44). FHA is known to be cross-reactive with species other than Bordetella, such as *C. pneumoniae* and *M. pneumoniae* and is therefore likely to be boosted more often (45). Prn is only expressed by the *Bordetella* genus and might have cross-reactive properties between different species of *Bordetella* (46). However, due to the immune pressure, circulating strains have changed during the years and a significant increase of Prn-negative *Bordetella* isolates was noticed around 2012 in Europe, leading to boost of immunity to other antigens than Prn (47, 48). This might also explain the best correlation found between vaccination and memory B cell frequencies for Prn since Prn is least affected by infection and therefore most directly affected by vaccination (49). Ptx specific memory B cell frequencies seem to be more elevated after infection than vaccination and remain more elevated even nine months post-infection (49). This indicates high stimulation of memory B cells to Ptx *in vivo* during an infection in contrast to vaccination, which might be explained by the altered immunogenicity caused by the chemically modified PT in aP vaccines (31). Vaccine responses against pertussis toxin might benefit from the inclusion of genetically detoxified Ptx instead of chemically detoxified Ptx in future vaccines (33). Memory B cell responses against native Ptx (as we measure it) are most interesting to study someone’s current immune status against *B. pertussis*, since Ptx is the most pathogenic product of this bacterium. However, Prn is probably most informative to study magnitude and duration of vaccine responses, since this is least affected by infection at present.

When we compared the participants divided into two categories based on the baseline memory B cell frequencies, we noticed significantly higher memory B cell frequencies specific to Ptx, FHA, and Prn in participants with > 1 memory B cell per 100,000 PBMCs at baseline, which can be interpreted as the presence of circulating memory B cells. As expected, antibody concentrations for Ptx and Prn were also significantly higher at all timepoints in individuals with circulating memory B cells at baseline. For FHA differences between the two categories were not significant at day 28 and 1 year which was expected considering the previously described correlation coefficient between memory B cells and antibody concentrations for this antigen. This interesting finding might be considered as a biomarker to assess the persistence of B cell memory following primary or booster pertussis vaccination, capable of producing a robust memory response among school-aged children, adolescents, and young and older adults. Another study, with a slightly different approach, found higher vaccine responses in participants with higher baseline antibody concentrations (50). Furthermore, similar results with baseline IgG-Ptx antibody concentrations have been observed for Ptx neutralising antibody titres (51).

The strengths of this study are in concurrently assessing B cell (re)activation in three countries in four age groups with identical clinical and laboratory methodologies. All protocols used for measuring memory B cell results were harmonised between the countries. Furthermore, the participants in this study were monitored for up to one year providing longitudinal data on memory B cell responses after an aP booster vaccination. In addition, all plates were measured centrally at the RIVM, which excludes the possibility of any result-based bias due to multi-site ELISpot measuring approaches. Limitations of this multicentre trial include the small number of adolescents included in the wP versus aP analysis, which may be reflected in the results between these two groups as we observed elevated, but not significantly higher, memory B cell frequencies for the wP primed participants.

To conclude, we showed that memory B cell frequencies are highly elevated after aP boosting and that frequencies were still elevated after one year. Furthermore, country-to-country variations were small indicating that responses to aP are similar regardless of the pertussis epidemiology in different populations. Our results also show that memory B cell frequencies are reduced in the older adults probably due to immunosenescence. Circulating memory B cells seem most pronounced during adolescence, which is probably a consequence of both vaccinations and natural boosting in this age group. Baseline presence of circulating memory B cells was found to predict the outcome of memory B cell responses after a booster dose. From this it can be deduced that, in order to induce a good booster response, it is important to have circulating memory B cells when a subsequent booster is administered. Therefore, circulating memory B cells might be a marker to determine intervals for boosting pertussis immunity.

## Data Availability Statement

Individual participant data that underlie the results reported in this article, have been de-identified and deposited in the central database of the PERISCOPE Consortium and can be accessed by a request to the PERISCOPE management team.

## Ethics Statement

This human clinical study was designed and conducted in accordance with the provisions of the Declaration of Helsinki (1996) and the International Conference on Harmonisation Guidelines for Good Clinical Practice. The trial was registered at the EU Clinical Trial database (EudraCT number 2016-003678-42) and was approved by the Medical Research Ethics Committees United (MEC-U, NL60807.100.17-R17.039) in the Netherlands, the South Central - Hampshire B Research Ethics Committee (REC, 19/SC/0368) in the UK, and the MREC UTU (ETMK Dnro: 129/1800/2017) in Finland. Written informed consent was obtained from all adult participants, and parents or legal guardian of minors, at the start of the study.

## Author Contributions

GB, DK and JM were responsible for the conception and design of the study with contribution of QH, A-MBu, ES, PV, A-MBa, MP. PV, A-MBa, MV, AK, SB, LR, JT, KS, HH, RL, EC, and M-lZ-B contributed substantially to the data acquisition. JK was responsible for the data analysis with contribution from A-MBa, PV, AK, and MV. PV, A-MBa, MV, A-MBu, DK, QH contributed substantially to the interpretation of the data. PV, A-MBa, MV wrote the first draft of the manuscript and A-MBu, DK, QH, GB, JM, JK, AK, SB, LR, JT, KS, HH, RL, EC, ES, and M-lZ-B revised it critically for important intellectual content. All authors provided approval for publication of the content and agreed to be accountable for all aspects of the work in ensuring that questions related to the accuracy or integrity of any part of the work are appropriately investigated and resolved.

## Funding

PERISCOPE has received funding from the Innovative Medicines Initiative 2 Joint Undertaking under grant agreement No 115910. This Joint Undertaking receives support from the European Union’s Horizon 2020 research and innovation programme and EFPIA and BMGF.

## Author Disclaimer

Results reflect the authors’ view, the Innovative Medicines Initiative 2 Joint Undertaking is not responsible for any use that may be made of the information it contains.

## Conflict of Interest

The authors declare that the research was conducted in the absence of any commercial or financial relationships that could be construed as a potential conflict of interest.

## Publisher’s Note

All claims expressed in this article are solely those of the authors and do not necessarily represent those of their affiliated organizations, or those of the publisher, the editors and the reviewers. Any product that may be evaluated in this article, or claim that may be made by its manufacturer, is not guaranteed or endorsed by the publisher.
