# Supplementary figures and images for: Memory B Cell Activation Induced by Pertussis Booster Vaccination in Four Age Groups of Three Countries
Source: Front Immunol. 2022 May 23;13:864674. doi: 10.3389/fimmu.2022.864674 (PMC9168128; doi:10.3389/fimmu.2022.864674)

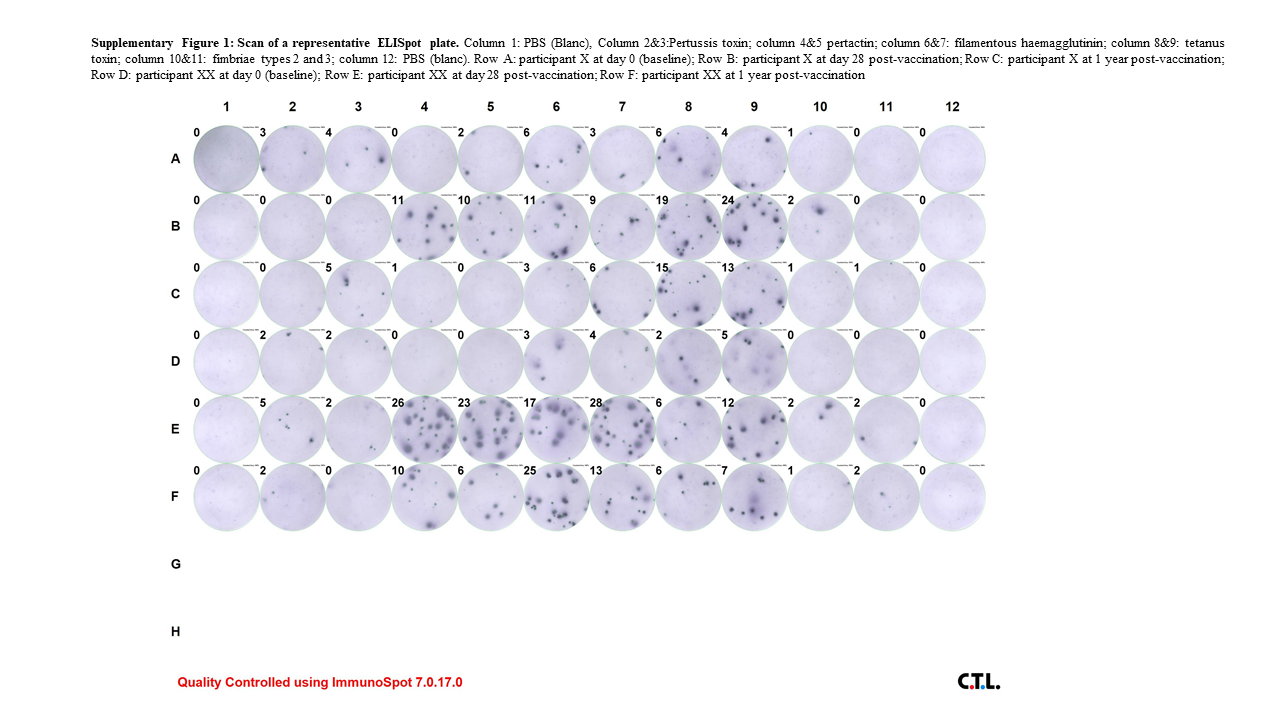

Supplement: Supplementary file 1 [file Image_1.tif]

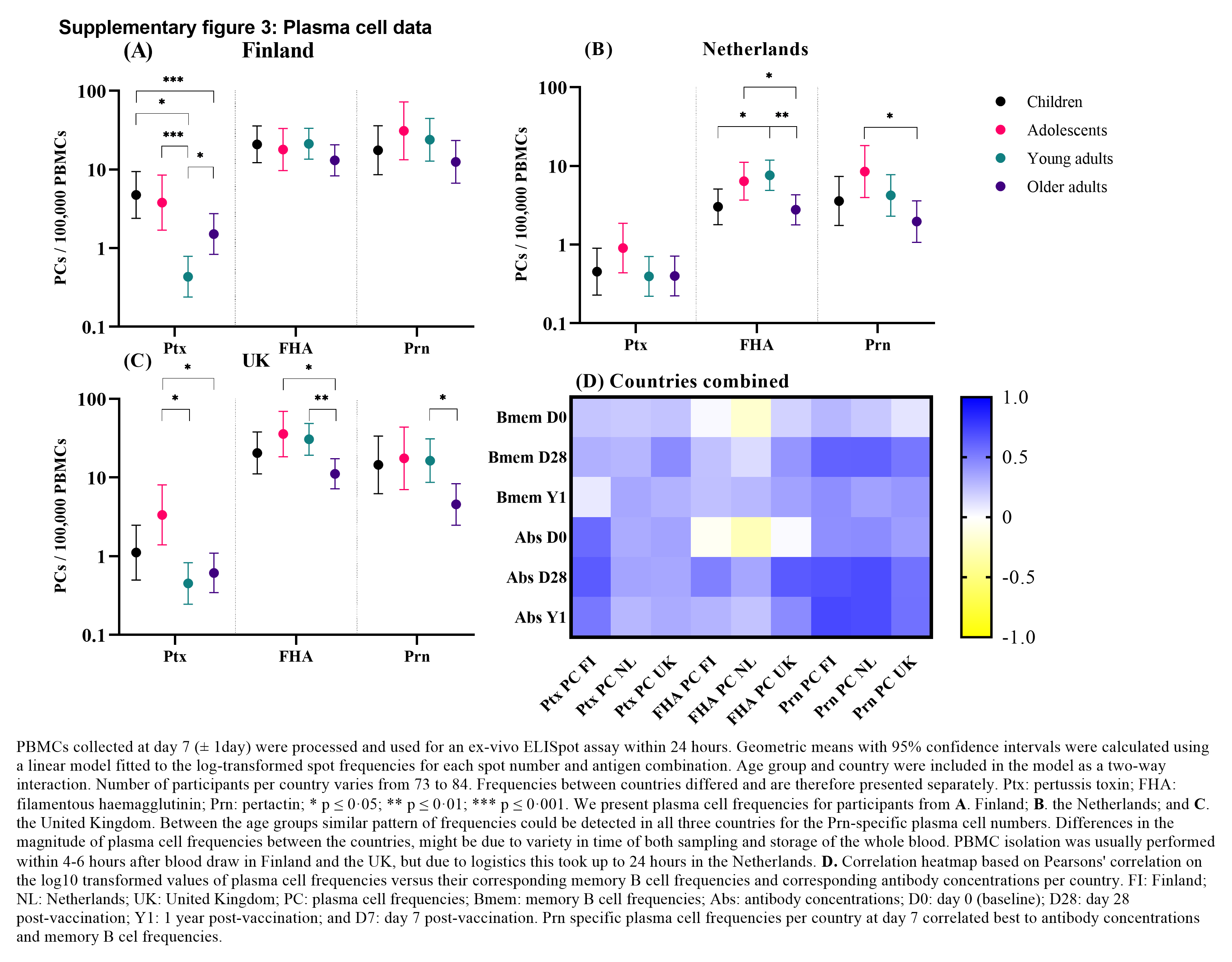

Supplement: Supplementary file 3 [file Image_3.tif]
