# Supplementary material for: Memory B Cell Activation Induced by Pertussis Booster Vaccination in Four Age Groups of Three Countries
Source: Front Immunol. 2022 May 23;13:864674. doi: 10.3389/fimmu.2022.864674 (PMC9168128; doi:10.3389/fimmu.2022.864674)
Supplement: Supplementary file 4 [file Table_1.docx]

**Supplemental Table 1: Antigen specific geometric mean spot frequencies per age group and country.** Geometric mean memory B cell spot frequencies with 95% confidence intervals from four age groups per country were calculated using a linear mixed model on the log10 transformed data taking the longitudinal structure into account. Ptx: pertussis toxin; FHA: filamentous haemagglutinin; Prn: pertactin; CI: 95% confidence interval.

| **Antigen** | **Timepoint** | **Country** | **Children** | **Adolescents** | **Young adults** | **Older adults** |
| --- | --- | --- | --- | --- | --- | --- |
| Ptx  GM spot frequencies (CI) in spots/100,000 PBMCs | Day 0 | FI | 0.3 (0.2-0.6) | 0.8 (0.4-1.9) | 0.2 (0.1-0.4) | 0.3 (0.2-0.5) |
|  |  | NL | 0.7 (0.4-1.5) | 0.8 (0.5-1.5) | 0.7 (0.3-1.4) | 0.7 (0.4-1.5) |
|  |  | UK | 0.7 (0.3-1.4) | 0.9 (0.5-1.7) | 0.4 (0.2-0.7) | 0.3 (0.1-0.5) |
|  | Day 28 | FI | 2.8 (1.4-5.7) | 5.4 (2.4-12) | 1.0 (0.6-1.9) | 1.2 (0.7-2.3) |
|  |  | NL | 4.7 (2.3-9.5) | 4.2 (2.4-7.4) | 4.6 (2.2-9.3) | 2.8 (1.3-5.7) |
|  |  | UK | 2.9 (1.4-6.0) | 5.1 (2.7-9.6) | 3.1 (1.7-5.8) | 2.2 (1.2-4.1) |
|  | 1 year | FI | 0.4 (0.2-0.9) | 0.8 (0.3-1.8) | 0.7 (0.4-1.2) | 0.3 (0.2-0.6) |
|  |  | NL | 1.0 (0.5-1.9) | 1.8 (1.0-3.1) | 1.7 (0.8-3.6) | 1.4 (0.7-2.8) |
|  |  | UK | 1.4 (0.7-2.9) | 2.1 (1.1-3.8) | 1.0 (0.5-1.9) | 1.1 (0.6-2.0) |
| FHA  GM spot frequencies (CI) in spots/100,000 PBMCs | Day 0 | FI | 1.5 (0.9-2.7) | 3.9 (2.0-7.7) | 0.5 (0.3-0.8) | 0.3 (0.2-0.5) |
|  |  | NL | 2.7 (1.5-4.8) | 1.6 (1.0-2.6) | 1.0 (0.6-1.9) | 1.7 (0.9-3.1) |
|  |  | UK | 2.4 (1.3-4.3) | 3.8 (2.3-6.4) | 0.8 (0.5-1.4) | 0.8 (0.5-1.3) |
|  | Day 28 | FI | 18 (9.9-31) | 18 (9.2-36) | 12 (7.2-19) | 3.5 (2.1-5.7) |
|  |  | NL | 15 (8.5-28) | 18 (11-29) | 22 (12-39) | 16 (8.6-28) |
|  |  | UK | 21 (12-38) | 28 (16-46) | 26 (15-43) | 12 (7.3-21) |
|  | 1 year | FI | 3.3 (1.8-5.8) | 9.9 (5.0-20) | 3.8 (2.3-6.2) | 1.7 (1.0-2.7) |
|  |  | NL | 3.7 (2.1-6.6) | 5.2 (3.3-8.4) | 3.1 (1.7-5.8) | 4.2 (2.3-7.5) |
|  |  | UK | 5.9 (3.2-11) | 5.9 (3.6-9.8) | 4.2 (2.4-7.1) | 2.0 (1.2-3.3) |
| Prn  GM spot frequencies (CI) in spots/100,000 PBMCs | Day 0 | FI | 0.4 (0.2-0.7) | 0.9 (0.4-2.0) | 0.3 (0.2-0.5) | 0.2 (0.1-0.4) |
|  |  | NL | 0.8 (0.4-1.6) | 0.5 (0.3-0.9) | 0.5 (0.2-1.0) | 0.4 (0.2-0.8) |
|  |  | UK | 0.8 (0.4-1.6) | 0.9 (0.5-1.7) | 0.3 (0.2-0.6) | 0.4 (0.2-0.8) |
|  | Day 28 | FI | 8.6 (4.4-17) | 23 (11-51) | 12 (7-21) | 1.7 (1.0-3.0) |
|  |  | NL | 8.0 (4.1-16) | 9.5 (5.5-16) | 4.5 (2.3-9.0) | 4.1 (2.1-8.2) |
|  |  | UK | 7.7 (3.9-15) | 8.9 (4.9-16) | 15 (8.1-27) | 3.9 (2.1-7.1) |
|  | 1 year | FI | 2.0 (1.0-4.0) | 4.8 (2.2-11) | 2.0 (1.1-3.5) | 0.6 (0.3-1.1) |
|  |  | NL | 1.5 (0.8-2.9) | 1.9 (1.1-3.3) | 1.2 (0.6-2.4) | 0.9 (0.5-1.8) |
|  |  | UK | 1.8 (0.9-3.6) | 2.3 (1.2-4.1) | 2.6 (1.4-4.9) | 1.0 (0.5-1.8) |
